# LESSONS LEARNED FROM THE SUMMER RESEARCH INSTITUTE/TRAINING NEEDS OF EARLY RESEARCHERS IN DEMENTIA

**DOI:** 10.1093/geroni/igae098.1123

**Published:** 2024-12-31

**Authors:** Sheryl Zimmerman, Sam Fazio

**Affiliations:** University of North Carolina at Chapel Hill (UNC), Chapel Hill, North Carolina, United States; Alzheimer’s Association, Chicago, Illinois, United States

## Abstract

Across the first three years of the Alzheimer’s Association Interdisciplinary Summer Research Institute (AA-ISRI), 98% of respondents indicated they were satisfied with the program, 95% felt the program increased their understanding of Alzheimer’s, and 90% said the program improved their independent research skills. Alongside the positive reception of AA-ISRI, feedback from participants, faculty, and faculty leadership led to several adjustments and changes to the structure and content of the program. For example, breakout sessions meant to provide deeper exposure to issues targeted to either public health or psychosocial research were found to be of relevance to both tracks of trainees, informing overlapping needs despite the particular area of focus. This and other changes have refined the overall flow of the program, the relevance of information shared, and the recruitment and selection of appropriate participants. This presentation will review the key lessons learned that have helped to shape the AA-ISRI over the first three years, future improvements that have been discussed for subsequent years of the program, and ideas and considerations for organizations looking to implement similar programs for researchers, and mentors working with individual mentees.

